# UMI-guided single locus sequence typing method for phylotyping *Cutibacterium acnes* from skin samples

**DOI:** 10.3389/fcimb.2026.1807759

**Published:** 2026-05-20

**Authors:** Elody Orcel, Erwin Sentausa, Hayat Hage, Karen Louis, May Taha, Samuel Bellais, Adrien Villain, Laurent Beloeil, Steven Sijmons, Nathalie Devos, Adrien Saliou

**Affiliations:** 1 BIOASTER, Microbiology Technology Institute, Lyon, France; 2 GSK, Rixensart, Belgium; 3 Lesaffre Institute of Science and Technology, Marcq-en-Barœul, France

**Keywords:** Amplicon sequencing, *Cutibacterium* (Propionibacterium) *acnes*, DNA extraction, library preparation, microbiome, phylotyping, skin, strip samples

## Abstract

**Introduction:**

*Cutibacterium acnes* is a dominant member of the human skin microbiota and displays substantial strain-level diversity with relevance for skin health and disease. However, accurate characterization of *C. acnes* lineages directly from skin samples remains challenging due to low biomass, host DNA contamination, and limitations of short-read sequencing.

**Methods:**

Here, we present SLST-Seq, a culture-independent approach based on single-locus sequence typing (SLST), enabling strain-level profiling of *C. acnes* from low-input skin-strip samples. SLST-Seq adapts the LUMI-Seq® synthetic long-read sequencing technology to the *C. acnes* SLST marker, combining unique molecular identifier barcoding with de novo assembly to reconstruct full-length SLST sequences with high accuracy.

**Result:**

Method performance was validated using single-isolate controls, defined genomic DNA mixtures, spike-in dilution series, and run-specific controls, demonstrating high specificity, quantitative accuracy across a wide range of target-to-background DNA ratios, and strong run-to-run reproducibility. Applied to skin-strip samples from healthy volunteers, SLST-Seq generated robust SLST profiles and revealed marked inter-individual variability, with donor-specific community structures largely conserved between face and back skin sites.

**Discussion:**

Overall, SLST-Seq provides a sensitive and scalable framework for in situ analysis of *C. acnes* population structure and supports high-resolution studies of skin microbiome composition from challenging clinical samples.

## Introduction

*Cutibacterium acnes* (formerly *Propionibacterium acnes*) is a dominant member of the human skin microbiota and one of the most abundant species in pilosebaceous follicles (Brüggemann et al., 2021). Although ubiquitous, *C. acnes* displays substantial genetic and functional diversity, with distinct phylogroups differing in host interaction and association with skin health and disease, particularly acne (Spittaels et al., 2020; Cobian et al., 2021; Yu et al., 2024). This diversity is increasingly recognized as a key determinant of whether *C. acnes* acts as a benign commensal or contributes to inflammatory pathology. Differences between lineages have been described at the level of virulence-associated factors, metabolic pathways, and host immune modulation. These lineages are commonly classified into six major phylogroups, IA1, IA2, IB, IC, II and III, based on multilocus sequence typing (MLST), for which two independent schemes have been described (Lomholt and Kilian, 2010; McDowell et al., 2011), and supported by the Single Locus Sequence Typing (SLST) scheme, where SLST groups A–E correspond to IA1, F to IA2, H to IB, G to IC, K to II and L to III (Scholz et al., 2014). While MLST infers population structure from sequence variation across multiple housekeeping loci, SLST achieves comparable phylogroup resolution from a single locus, making it more scalable for population-level studies. This stable correspondence between MLST and SLST has made the SLST locus a widely adopted proxy for population structure in both culture-based and culture-independent studies.

Accurate resolution of *C. acnes* lineages in skin microbiome samples remains challenging. Skin DNA is often low-biomass, degraded, and contaminated with host material, which can impair long-amplicon sequencing methods and complicate quantitative interpretation (Chen et al., 2023; Zhang et al., 2024). Moreover, sampling methods such as skin stripping or swabbing may recover uneven and sparse bacterial DNA, further increasing stochastic effects during amplification (Chen et al., 2023). The SLST scheme provides an informative single-locus marker for differentiating lineages, but conventional amplicon sequencing approaches may suffer from amplification bias in low-biomass samples (Markey et al., 2025), limiting quantitative accuracy and reproducibility across studies. Culture-based multiplex PCR phylotyping classifies isolates into phylogroups based on the presence or absence of phylogroup-discriminatory amplicons (Barnard et al., 2015), but is labor-intensive, selective and may underestimate *in situ* diversity (Petersen et al., 2017; Dagnelie et al., 2018b). In addition, cultivation may favor fast-growing or oxygen-tolerant strains, thereby distorting the relative abundance of phylogroups. Together, these limitations highlight the need for culture-independent, quantitatively accurate approaches for direct SLST profiling of complex skin samples.

To address these limitations, we adapted BIOASTER’s LUMI-Seq technology, which was originally developed for high-accuracy full-length sequencing of the bacterial 16S ribosomal RNA gene using unique molecular identifiers (UMIs), to the SLST locus (Gravdal et al., 2023; Roume et al., 2023). This synthetic long-read approach enables high-fidelity recovery of long amplicons from Illumina short-read sequencing, while substantially reducing PCR and sequencing errors. Therefore, the approach is well suited for low-input, degraded DNA typical of skin samples. Applying this strategy to SLST enables full-length reconstruction of the locus while preserving quantitative information across samples. Here, we applied this SLST-Seq workflow to characterize *C. acnes* phylotype composition in facial and back skin strip samples collected from healthy volunteers. Although facial skin has been examined in previous studies (Dagnelie et al., 2018a; Ahle et al., 2022; Feidenhansl et al., 2024), high-resolution paired comparisons of facial and back *C. acnes* communities at the phylotype or strain level remain limited. By combining a validated SLST sequencing workflow with paired anatomical sampling, this study aims to provide both methodological validation and new insights into the spatial organization of *C. acnes* populations on human skin.

## Results

**Figure 1:**
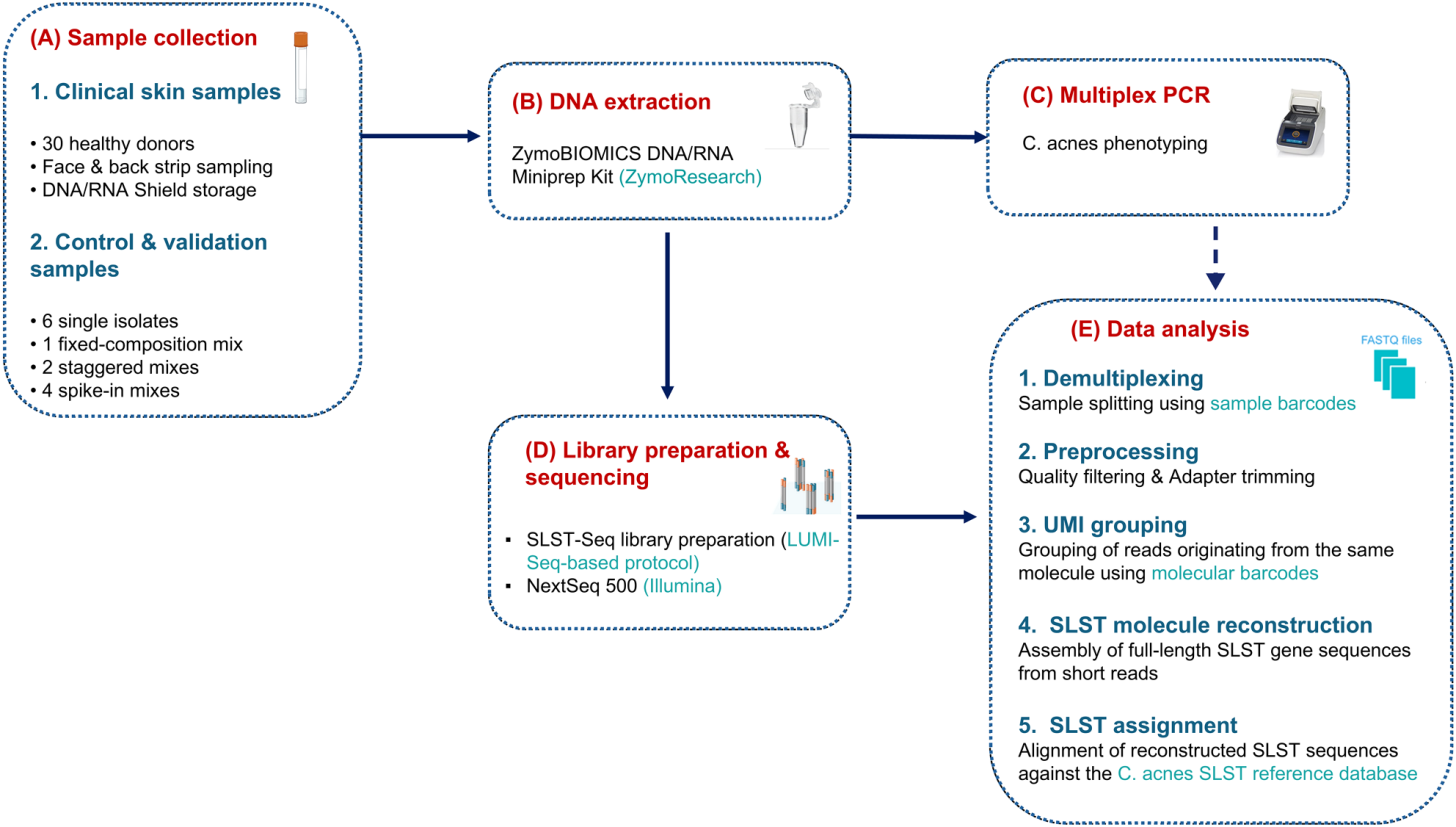
Overview of the study design and analytical workflow. **(A)** Skin-strip samples were collected from 30 healthy volunteers (face and back). In parallel, DNA-based control samples were included, comprising single-isolate controls, defined DNA mixtures, spike-in dilution series, and run-specific mock and negative controls. **(B)** Following DNA extraction, *Cutibacterium acnes* lineages were characterized using multiplex PCR **(C)** and SLST amplification **(D)**. Libraries were prepared using an Illumina-compatible SLST-Seq workflow and sequenced on a NextSeq 500 platform **(D)**. Sequencing data were processed through a dedicated bioinformatic pipeline to reconstruct full-length SLST sequences, assign SLST types, and generate quantitative profiles for downstream analyses **(E)**.

The overall study design, including sample collection and control types, is summarized in **Figure 1**, and a schematic overview of the SLST-Seq workflow is provided in **Supplementary Figure 1**. Briefly, the study included 13 DNA-based control samples comprising single-isolate preparations, defined DNA mixtures, and serial dilution series, as well as skin-strip samples from 30 healthy donors. The control samples were used to validate SLST-Seq performance, and the method was then applied to the clinical skin-strip samples. Detailed experimental and bioinformatic procedures are described in the Methods section.

### SLST-Seq assigns sequences to the correct SLST type with high specificity

**Figure 2:**
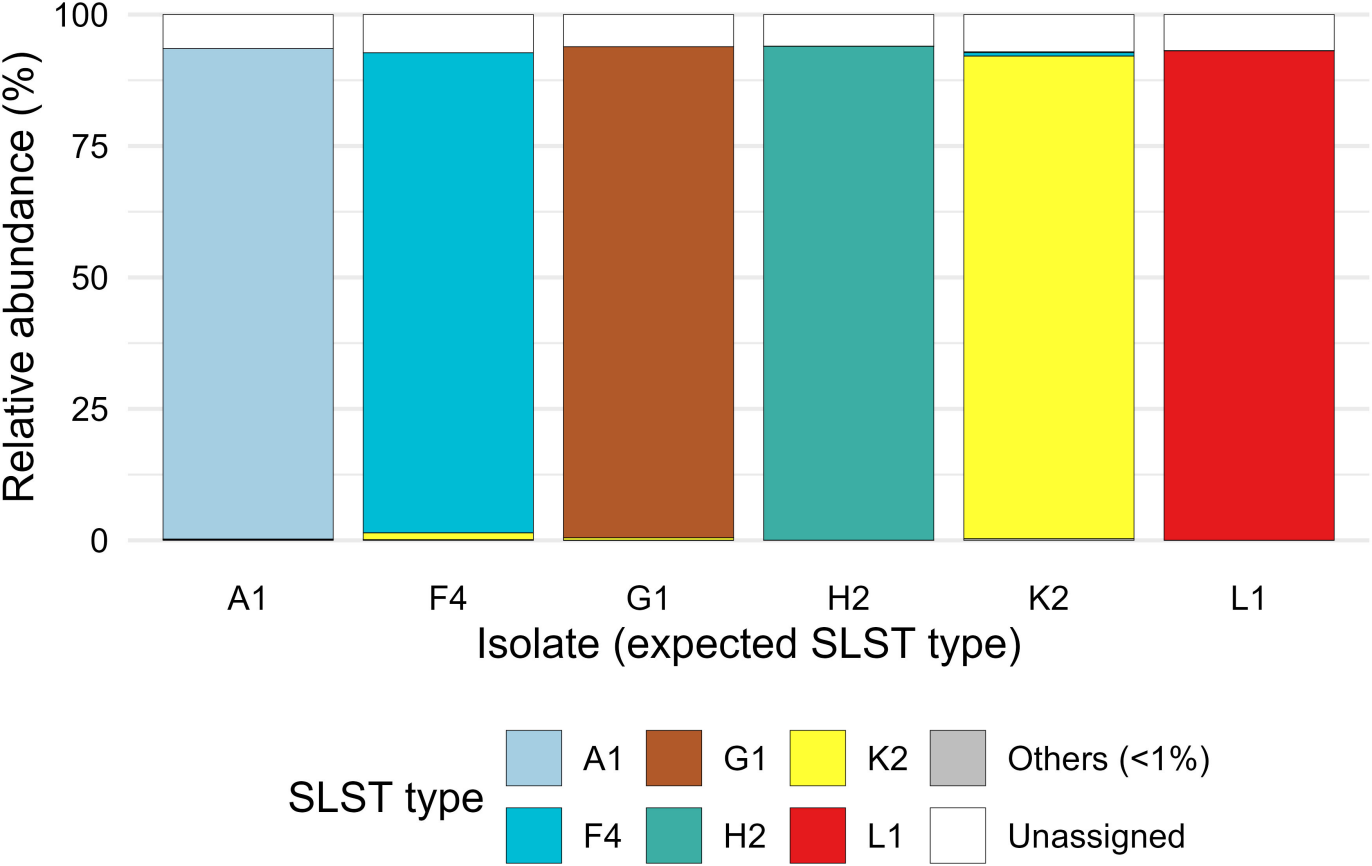
Specificity of the SLST assay on individual *C. acnes* isolates. Relative abundances of SLST types recovered from six isolates (A1, F4, G1, H2, K2 and L1). For each isolate, the expected outcome was the exclusive detection of its corresponding SLST type. The majority of sequences were correctly assigned to the expected type, with only minor fractions assigned as “Unassigned” or to other SLST types.

To assess assay specificity, six *C. acnes* isolates were analyzed individually, one per major phylogroup: SLST types A1 (IA1), F4 (IA2), G1 (IC), H2 (IB), K2 (II) and L1 (III). For each isolate, the expected outcome was the exclusive recovery of its corresponding SLST type. Across isolates, an average of 93% of sequences were correctly assigned to the expected SLST type, with the remaining distributed between “Unassigned” and a small fraction of other SLST types (**Figure 2**). These results confirm that the SLST assay is highly specific, reliably assigning sequences to the correct SLST type when tested on single-isolate controls.

### SLST-Seq accurately quantifies SLST type composition in defined DNA mixtures

All mock communities used for the validation of the SLST assay (**Supplementary Table 1**) were processed with the full-length SLST workflow. Across the three defined mixes (FixedMix, StaggeredMix-L1 and StaggeredMix-G1), between 7,043 and 8,192 full-length SLST sequences per sample were reconstructed, with ~93–96% of total reads assigned to SLST types and essentially all of those assignments corresponding to the expected types (**Table 1**).

**Table 1:** Sequencing output and assignment metrics for defined *C. acnes* DNA mixes used to validate the SLST assay.

Mix	Sequenced read pairs (M)	Full-length SLST sequences reconstructed	Sequences assigned to any SLST type	Sequences assigned to expected SLST types	% assigned (of total)	% assigned expected (of total)	Bray-curtis similarity (observed vs. expected)
FixedMix	1.1	8192	7639	7634	93.2	93.2	0.939
StaggeredMix-L1	1.2	7732	7368	7367	95.3	95.3	0.976
StaggeredMix-G1	1.1	7043	6739	6739	95.7	95.7	0.983

**Figure 3:**
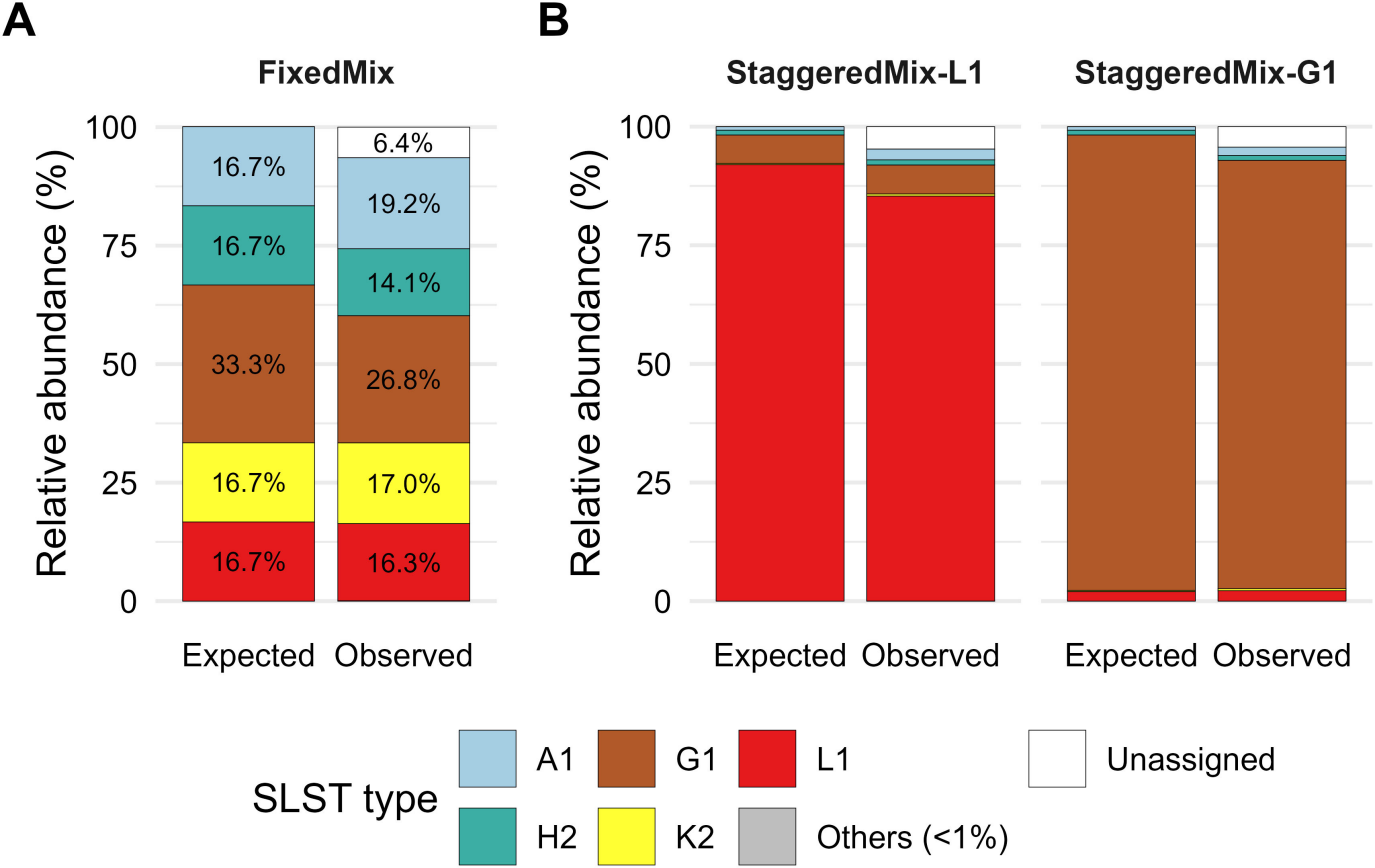
Expected and observed SLST composition of defined *C. acnes* DNA mixes. **(A)** Fixed composition mix (FixedMix). Stacked bars show the theoretical (Expected) and measured (Observed) relative abundances of five SLST types (A1, G1, H2, K2 and L1) plus any residual reads (“Others” <1%) and unassigned sequences. **(B)** Highly imbalanced controls. Stacked bars show the expected and observed compositions for StaggeredMix-L1 (92% L1, ≤6% others) and StaggeredMix-G1 (96% G1, ≤2% others). The assay correctly recovers the dominant type in each mixture with only minor deviations from the theoretical composition.

In the fixed-composition community (FixedMix; 33.3% G1 and 16.7% each of A1, H2, K2 and L1), the observed relative abundances matched the theoretical composition for most types (**Figure 3A**), though G1 was recovered below its expected proportion (26.4% vs expected 33.3%). This demonstrates that the assay yields reproducible quantitative data for a non-even but constant mixture of SLST types.

In the highly imbalanced communities, the SLST assay correctly identified the dominant type and detected the minority types at their expected low frequencies. In StaggeredMix-L1 (92% L1, ≤6% others) and StaggeredMix-G1 (96% G1, ≤2% others), the recovered proportions reflected the intended dominance of L1 or G1 with only minor deviation from the target values (**Figure 3B**). Bray-Curtis similarity between observed and expected compositions ranged from 0.939 to 0.983 across the three defined mixes (**Table 1**), confirming high quantitative accuracy across a range of input compositions.

### SLST-Seq yields reproducible profiles across independent sequencing runs

An internal “Mock” control consisting of six SLST types (A1, F4, G1, H2, K2 and L1) at equal nominal proportions and spiked with *E. coli* DNA (2:98 mass ratio) and a negative control (Nuclease-free water) were included in every sequencing run to monitor assay performance.

Across twelve independent sequencing runs, the relative abundances of the six target SLST types in the Mock control remained highly consistent (**Supplementary Figure 2A**), with only minor fluctuations between runs. The percentage of reads assigned to sequences other than the six intended SLST types (“Others”) stayed close to zero, while the fraction of unassigned reads was low and stable (generally <10% of total reads) across all runs (**Supplementary Figure 2B**). These results demonstrate that the SLST workflow yields reproducible quantitative profiles and low background across multiple sequencing runs. In addition to run-to-run reproducibility, we assessed the quantitative agreement between observed and expected proportions of the six SLST types in the Mock control (equal nominal fractions of 16.7% each). The average relative abundances recovered over twelve runs were very close to the expected values, with only minor deviations (**Table 2**). In particular, A1 (19.4%) and F4 (18.0%) tended to be slightly overrepresented, while G1 (13.9%), H2 (15.6%) and L1 (15.7%) were slightly underrepresented. K2 showed the smallest deviation from its expected proportion (17.5% observed vs. 16.7% expected). Variability across runs remained low, with standard deviations between 1.2% and 1.9% for all six types (**Supplementary Table 2**). These results confirm that the assay provides consistent and quantitative recovery of the targeted SLST types in repeated control experiments.

**Table 2:** Expected and mean observed relative abundances of the six SLST types in the Mock control.

SLST type	Expected proportion (%)	Mean observed proportion (%)
A1	16.7	19.4
F4	16.7	18.0
G1	16.7	13.9
H2	16.7	15.6
K2	16.7	17.5
L1	16.7	15.7

The Mock control was designed to contain equal fractions (16.7%) of six SLST types (A1, F4, G1, H2, K2 and L1). Values shown are the mean observed proportions across 12 sequencing runs.

In the twelve negative controls processed across runs, the maximum number of full-length SLST sequences detected was 50 (**Supplementary Table 3**). This value was therefore used as the background noise threshold: samples yielding fewer than 50 sequences were considered outliers and excluded from downstream analyses.

### Quantitative accuracy is maintained at low *C. acnes* DNA fractions

**Figure 4:**
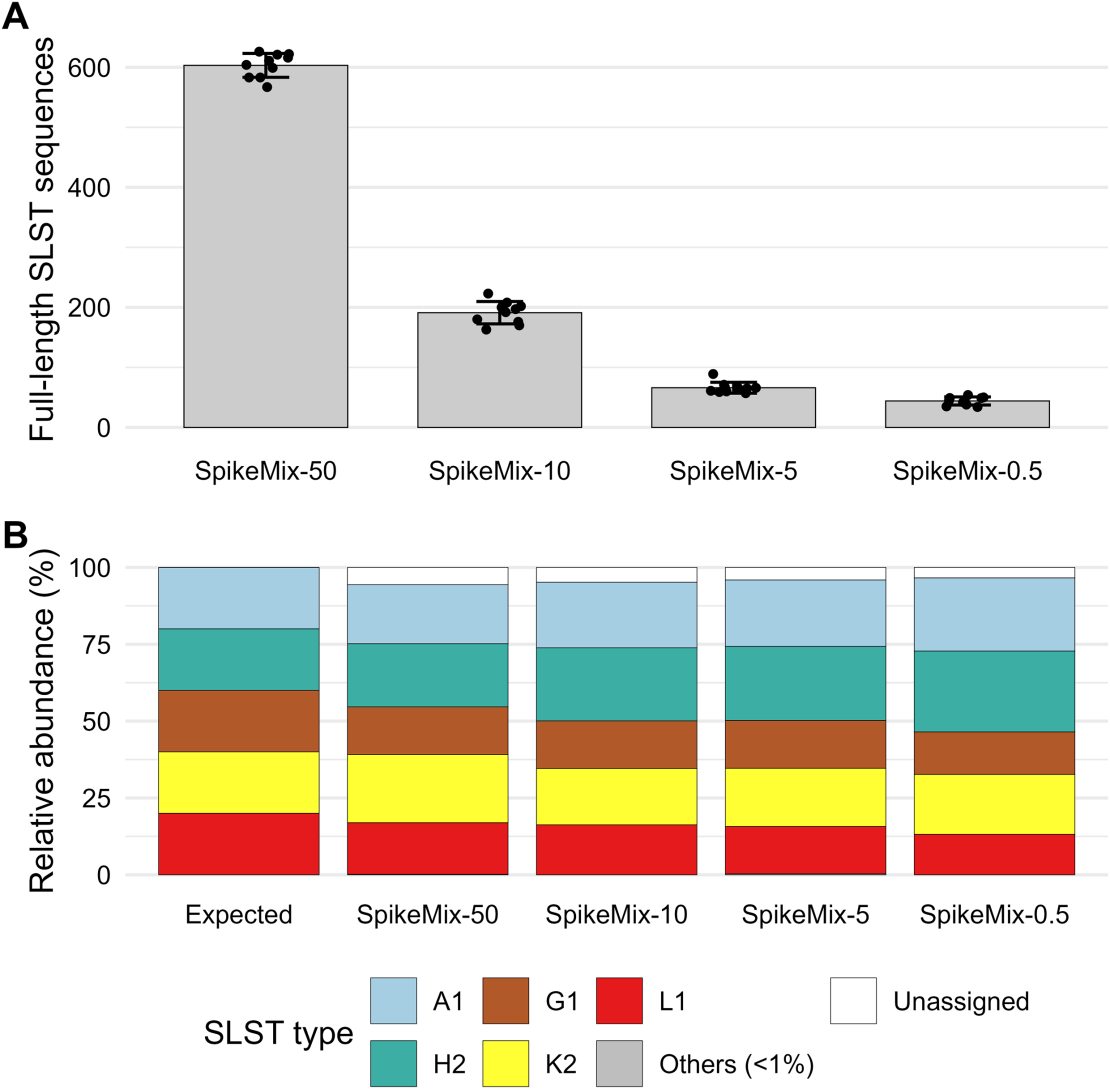
Performance of the SLST assay on spike-in mixtures of *C. acnes* DNA with increasing background DNA. **(A)** Total number of full-length SLST sequences reconstructed from spike-in mixtures containing decreasing proportions of *C. acnes* genomic DNA (SpikeMix-50, -10, -5, and -0.5) supplemented with *E. coli* DNA. Bars represent mean counts across replicates (± SD), and dots indicate individual replicates. **(B)** Relative abundances of SLST types recovered from the same spike-in mixtures, shown alongside the theoretical composition (Expected, left). Stacked bars illustrate the five targeted SLST types (A1, G1, H2, K2, L1), with residual reads grouped as “Others (< 1%)” and “Unassigned.” All SpikeMix samples were processed without pre-amplification, using 2 µL of purified DNA at 0.125 ng/µL (total input 0.25 ng) directly into the molecular barcoding step, yielding absolute *C. acnes* DNA inputs of 125 pg (SpikeMix-50), 25 pg (SpikeMix-10), 12.5 pg (SpikeMix-5), and 1.25 pg (SpikeMix-0.5).

To evaluate assay performance under low target-DNA conditions, *C. acnes* DNA was diluted with increasing amounts of *E. coli* genomic DNA (SpikeMix-50 to SpikeMix-0.5). As the proportion of target DNA decreased, the total number of reconstructed full-length SLST sequences declined accordingly (**Figure 4A**). Despite this reduction in sequencing depth, relative abundances of the five SLST types in the spiked mixes remained close to the expected 20% each (**Supplementary Table 1**), even at the lowest input tested (0.5% *C. acnes* DNA; **Figure 4B**). All five SLST types were detected above the background threshold at all dilution levels, including SpikeMix-0.5 (0.5% *C. acnes* DNA, corresponding to approximately 1.25 pg absolute input at a total DNA input of 0.25 ng). Bray-Curtis similarity to the expected composition decreased modestly with decreasing *C. acnes* DNA fraction, from 0.937 ± 0.024 at 50% to 0.834 ± 0.052 at 0.5% (**Supplementary Table 4**), reflecting increased stochastic variability at the lowest input fraction. These results indicate that the assay maintains quantitative accuracy under conditions of low target abundance and high background DNA.

### Clinical skin-strip samples reveal donor-specific *C. acnes* community structures with partial conservation between face and back

After validation on defined mixes and controls, the SLST workflow was applied to clinical skin-strip samples from 30 healthy volunteers (face and back). Across all 60 samples, sequencing yielded an average of 4,004 full-length SLST sequences per sample. Samples with fewer than 50 reconstructed sequences were excluded, consistent with the negative control threshold. Overall, 48 of 60 samples (80%) passed the threshold. In four donors, both face and back samples were excluded. An additional four excluded samples came from the back only, leaving the corresponding face samples available. The final dataset comprised 25 face and 23 back samples, including 22 paired donors, three donors with face samples only, and one donor with a back sample only.

**Figure 5:**
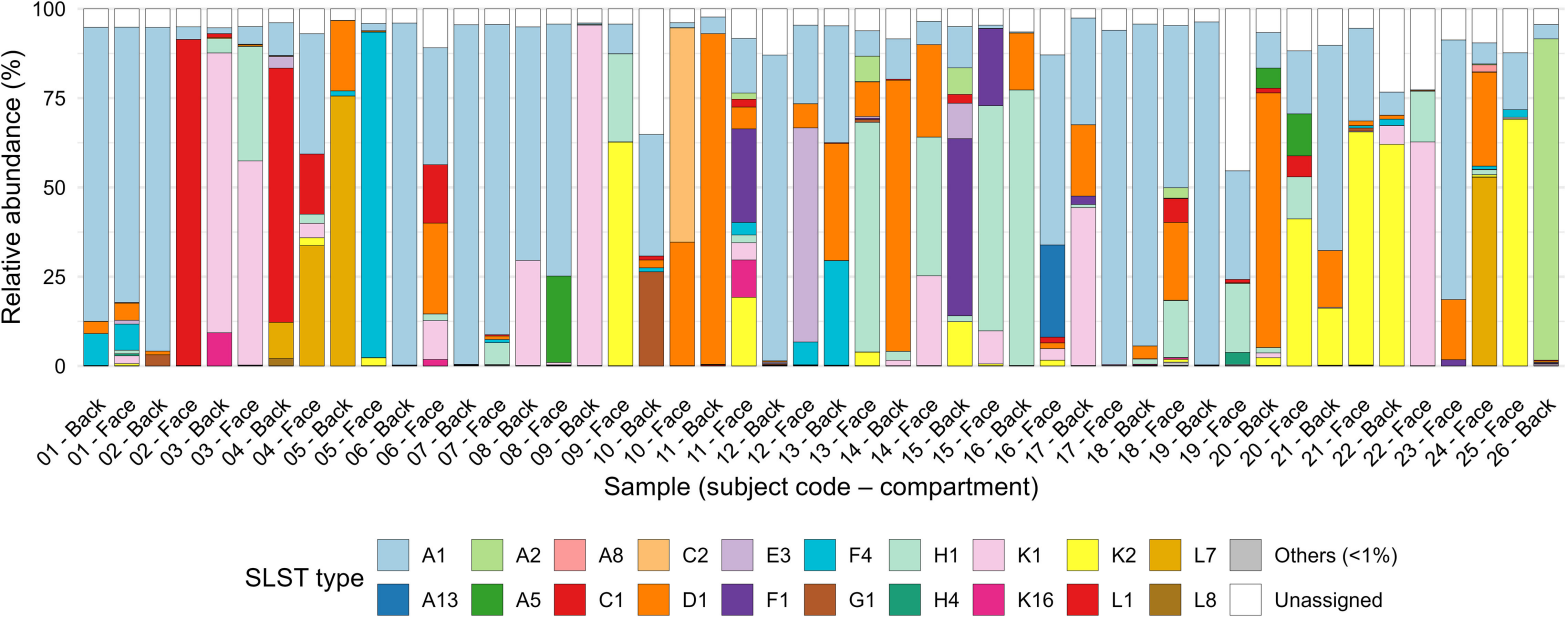
SLST-type composition across all face and back samples. Relative abundances of SLST types in all 48 samples that passed quality filtering (25 face and 23 back). Bars are grouped by donor, showing 22 paired donors, three face-only donors, and one back-only donor. Face and back samples from the same individual show broadly concordant SLST-type profiles, though some donors display markedly different dominant types between sites.

Face samples showed heterogeneous *C. acnes* community structures (**Figure 5**). A1 was the dominant SLST type in most donors, although its relative abundance varied considerably. Secondary types such as K2, D1, H1 and K1 were also detected at variable levels. Rare types (“Others”) were negligible, and unassigned sequences accounted for less than 10% in nearly all samples.

When considering all samples that passed quality filtering (n = 48), donor-specific community profiles were observed across both face and back (**Figure 5**). Among the 22 paired donors, face and back samples showed broadly concordant SLST-type profiles, with community composition more consistent within individuals than between different individuals, though some donors displayed markedly different dominant types between sites. Bray-Curtis dissimilarity between face and back samples within the same donor (mean = 0.652) was lower than between donors (mean = 0.724), though this difference did not reach statistical significance (Mann-Whitney U test, p = 0.070). To further characterize site-specific differences at the individual level, we performed paired Wilcoxon signed-rank tests comparing face and back relative abundances for each SLST type detected in at least five paired donors, with Benjamini-Hochberg correction for multiple testing. The strongest trend was observed for H1, which was enriched on the face relative to the back (mean 13.5% vs 4.1%, p = 0.005 uncorrected, p = 0.094 after correction), and for D1, which tended to be more abundant on the back (mean 15.9% vs 6.4%). A1, the most frequently detected type, also tended to be more abundant on the back (mean 40.8% vs 29.1%). However, no SLST type reached statistical significance after correction.

**Figure 6:**
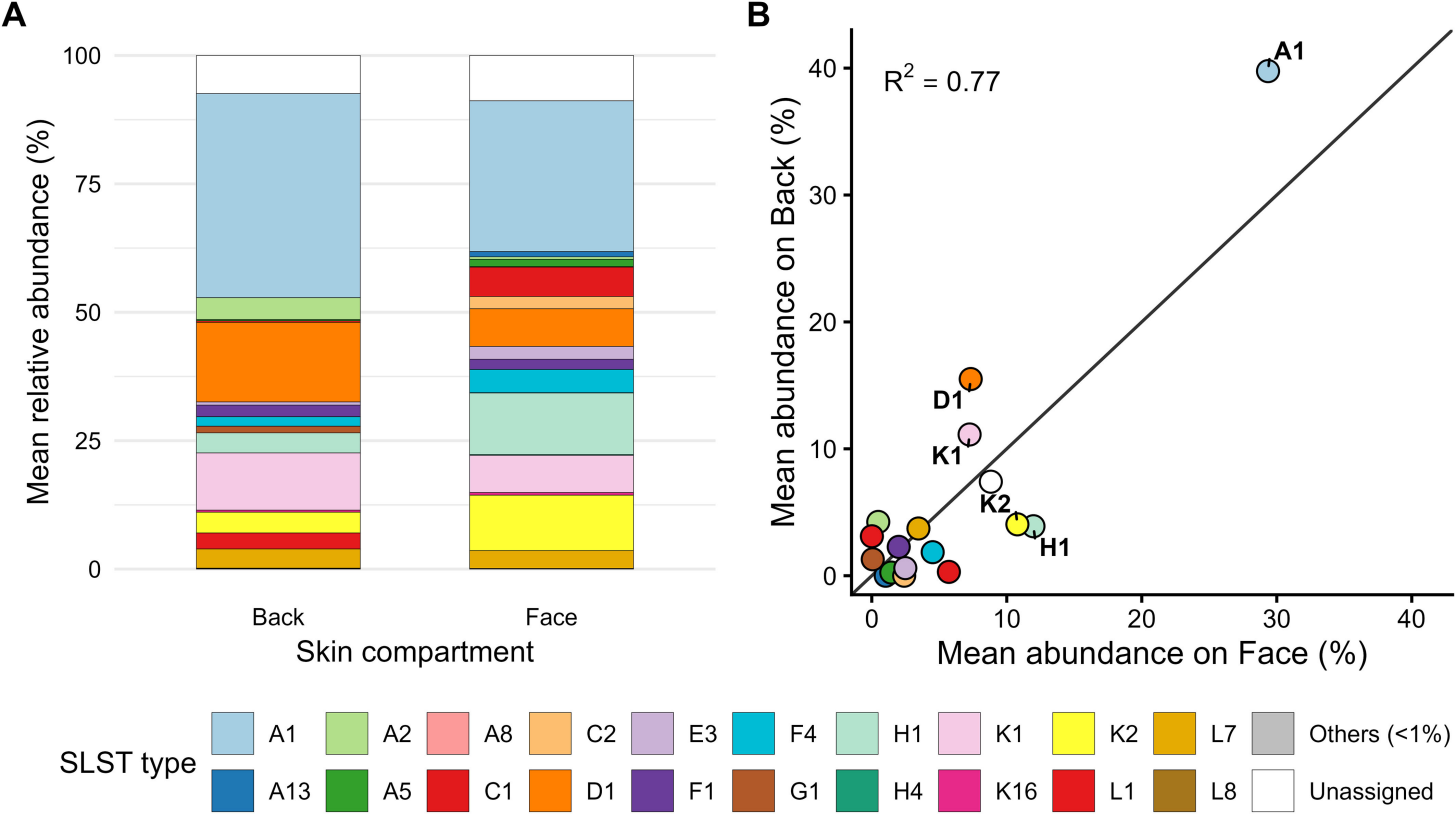
Distribution of *C. acnes* SLST types across skin compartments. **(A)** Mean relative abundances of SLST types across all face (n = 25) and back (n = 23) samples. *C. acnes* type A1 dominated both sites, representing 29.4% of the mean community on the face and 39.8% on the back. Among secondary types, K2 (10.8% vs. 4.0%) and H1 (12.0% vs. 3.9%) were enriched in face samples, whereas D1 (15.5% vs. 7.3%) and K1 (11.1% vs. 7.3%) were more abundant on the back. **(B)** Correlation of mean SLST-type abundances between face and back samples. Each point represents the mean relative abundance of an SLST type across the two skin sites (x-axis: face; y-axis: back). The diagonal line denotes equal abundance between sites. Mean community profiles were strongly correlated (R² = 0.77), with a few types showing site-specific enrichments.

When averaged across donors, both face and back samples were dominated by A1, representing 29.4% and 39.8% of the mean community composition, respectively (**Figure 6A**). Among the secondary types, K2 (10.8% in face vs. 4.0% in back) and H1 (12.0% vs. 3.9%) were enriched in face samples, whereas D1 (15.5% vs. 7.3%) and K1 (11.1% vs. 7.3%) were more abundant in back samples. Other SLST types were consistently detected at lower mean abundances.

At the community level, mean SLST-type abundances in face and back samples were strongly correlated (R² = 0.77; **Figure 6B**). Most types fell close to the diagonal, indicating broadly similar distributions across sites. Notably, H1 and K2 showed higher mean abundances on the face, while D1 and K1 showed higher mean abundances on the back, consistent with the trends identified in the paired individual-level analysis.

### SLST-Seq and multiplex PCR show concordant phylotype-level detection with quantitative differences

Phylotype-level abundances obtained by multiplex PCR and by SLST sequencing were compared across seven back skin samples from healthy individuals (**Table 3**). Both methods identified phylotype IA1 as the most frequent lineage, although the relative proportions differed substantially between approaches; Pearson *r* = 0.315, mean absolute error (MAE) = 19.6% across all phylotypes and samples (**Supplementary Figure 3**). Other phylotypes were variably detected, and discrepancies were particularly evident for low-abundance phylotypes and for those detected by only one of the two methods in a given sample. This comparison should be interpreted as an exploratory assessment of phylotype-level concordance rather than a formal benchmarking exercise, as the two methods characterize different biological fractions of the community.

**Table 3:** Comparison of *C. acnes* phylotype relative abundances obtained by multiplex PCR and SLST.

Sample	Method	N clones/sequences	Percentage of IA1 (%)	Percentage of IA2 (%)	Percentage of IB (%)	Percentage of IC (%)	Percentage of II (%)	Percentage of III (%)
01 - Back	PCR	29	6.9	0	0	3.4	89.7	0
01 - Back	SLST	1833	85.9	8.9	0	0	0	0
02 - Back	PCR	4	75	0	0	0	25	0
02 - Back	SLST	192	91.7	0	0	3.1	0	0
03 - Back	PCR	30	100	0	0	0	0	0
03 - Back	SLST	1205	3	0	4.1	0	87.6	0
04 - Back	PCR	33	0	0	0	0	100	0
04 - Back	SLST	8268	12.7	0	0	0	0	83.3
05 - Back	PCR	2	0	100	0	0	0	0
05 - Back	SLST	9581	19.8	1.4	0	0	0	75.6
06 - Back	PCR	40	100	0	0	0	0	0
06 - Back	SLST	4665	95.9	0	0	0	0	0
07 - Back	PCR	42	90.5	0	0	0	9.5	0
07 - Back	SLST	2404	95.4	0	0	0	0	0

For each of the seven *C. acnes*–positive skin samples analyzed by both methods, the table reports the number of *C. acnes* colonies (for multiplex PCR) or assembled SLST sequences (for SLST) used to estimate phylotype proportions. Relative abundances (%) are shown for the six main phylotypes (IA1–III).

## Discussion

This study demonstrates the relevance of applying targeted SLST sequencing to resolve *C. acnes* community structure directly from complex, low-biomass skin samples. By adapting a UMI-based synthetic long-read approach to the SLST locus, we provide a robust, sensitive, and reproducible workflow capable of quantitative strain-level profiling while avoiding culture-associated biases and limitations of short-read amplicon sequencing. This performance was supported by validation on single isolates, defined DNA mixtures, dilution series, and repeated run controls, demonstrating quantitative accuracy and stability across a range of input conditions. Together, these controls provide strong evidence that the observed lineage distributions in clinical samples reflect biological signal rather than technical noise.

Placed in the context of previous work that has included facial skin but rarely combined facial and dorsal sampling with high-resolution lineage typing (Dagnelie et al., 2018a; Ahle et al., 2022; Feidenhansl et al., 2024), our paired analysis provides direct insight into the spatial organization of *C. acnes* communities. The largely concordant SLST-type profiles observed between face and back within individuals indicate that host-specific factors play a dominant role in shaping lineage composition. Such factors may include host genetics, immune responsiveness, sebum composition, and long-term colonization history. At the same time, consistent site-associated enrichments of specific SLST types highlight the influence of local skin microenvironments, demonstrating that high-resolution, paired sampling is necessary to capture both shared and site-specific features of *C. acnes* population structure.

The face represents a particularly relevant anatomical niche, characterized by high follicular density, increased sebaceous activity, and greater environmental exposure (Spittaels et al., 2020; Brüggemann et al., 2021). These factors may modulate nutrient availability, oxygen tension, and exposure to external stressors, potentially influencing lineage-level fitness. Differences in sebum composition, oxygen availability, and exposure to cosmetic or environmental stressors could impose selective pressures on coexisting *C. acnes* lineages, though this remains to be formally demonstrated. The observed site-associated differences underscore the importance of including facial sampling when investigating *C. acnes* population structure, as restricting analyses to a single anatomical site may lead to incomplete or biased interpretations of strain-level diversity within individuals. This is particularly relevant for studies aiming to link microbial composition to dermatological phenotypes.

Our observations underscore the importance of resolving *C. acnes* phylogroups, which have been previously associated with distinct clinical contexts. In particular, lineages within phylogroups IA1 and IA2 have been frequently linked to inflammatory acne, whereas phylogroups IB and II have been reported in deep-tissue or device-related infections, and phylogroup III in spinal disc disease (Scholz et al., 2014; Spittaels et al., 2020; Brüggemann et al., 2021). By providing higher resolution than conventional phylotyping approaches, SLST-Seq represents a powerful tool to further explore how strain-level diversity relates to anatomical niches and clinically relevant contexts described in the literature. We note that the comparison with culture-based multiplex PCR presented here does not constitute a formal benchmark of SLST-Seq, as the two methods profile fundamentally different fractions of the community.

Beyond the present study, the SLST-Seq workflow opens perspectives for complementary developments, including targeted or digital PCR assays informed by SLST-derived sequence variation, as well as multiplexed strategies combining full-length 16S rRNA gene sequencing with SLST within a single workflow. Such approaches could facilitate rapid screening while preserving strain-level resolution in large-scale or longitudinal studies.

This study has several limitations. The cohort size was moderate and restricted to healthy volunteers, which limits extrapolation to disease contexts. The number of paired donors with samples from both sites passing quality filtering (n = 22) also limited statistical power for formal detection of site-specific SLST type enrichment at the individual level. In addition, SLST remains a single-locus marker and does not capture the full genomic diversity of *C. acnes*, as previously discussed (Scholz et al., 2014; Brüggemann et al., 2021). We also note that SLST type L (corresponding to phylotype III), included in control experiments to represent the full SLST sequence space, has since been reclassified as *Cutibacterium modestum* (Dekio et al., 2021); however, this type was not detected in any clinical sample and does not affect the conclusions of this study. Furthermore, a consistent underrepresentation of G1 was observed across both the FixedMix (26.4% vs expected 33.3%) and the run-level Mock control (mean 13.9% vs expected 16.7%; **Table 2**), suggesting a modest amplification bias against this SLST type in the multiplex PCR step. Nevertheless, SLST represents a pragmatic compromise between resolution, scalability, and cost-effectiveness for population-level studies. The conservative background threshold applied may exclude samples with extremely low bacterial loads. Finally, a direct benchmarking of SLST-Seq against conventional paired-end 300 bp SLST amplicon sequencing was not performed, and would be valuable to formally assess the extent to which UMI-based consensus assembly reduces amplification bias and sequencing errors relative to conventional approaches. Future studies integrating additional loci or whole-genome approaches could further refine strain-level resolution.

In conclusion, SLST-Seq provides a robust and scalable approach for high-resolution profiling of *C. acnes* lineages in skin microbiome samples. By combining methodological rigor with direct application to paired facial and dorsal samples, this work contributes both a validated analytical framework and new biological insights into the spatial organization of *C. acnes* populations on human skin.

## Methods

### Collection of skin samples

Thirty healthy volunteers were included in the study, with samples collected equally from the face and the back. Volunteers were recruited at two clinical centers in France: Dermscan (Villeurbanne) and LyREC (Lyon), following a preselection visit and written informed consent. Eligible participants were aged 18–25 years, had no history of skin pathology, and had not received systemic antibiotics, antivirals, or anti-inflammatory treatments in the month prior to sampling. Participants were instructed not to apply make-up or clean the sampling zones in the 12 hours before sampling. Skin samples were obtained using the 3S Biokit adhesive strip system and preserved in a DNA/RNA stabilization solution (DNA Shield, Zymo Research). Samples were shipped at 4 °C to BIOASTER (Lyon, France) for downstream molecular analyses.

### Preparation of controls

Before applying the SLST assay to clinical skin samples, we first evaluated its performance on defined genomic-DNA mixtures of *C. acnes*. Five SLST types were selected (A1, G1, H2, K2 and L1) representing the major phylogroups (phylotypes IA1, IC, IB, II and III, respectively); we note that the lineage corresponding to SLST type L (phylotype III) has since been reclassified as *Cutibacterium modestum* (Dekio et al., 2021), but was included here to span the full range of SLST locus sequence diversity. Genomic DNA for A1 was obtained from DSMZ-1897, for H2 from DSMZ-16379, and the remaining SLST types were sourced from clinical isolates. DNA concentrations were determined with the QuantiFluor One dsDNA kit (Promega, E4871) before mixing.

Three defined DNA mixtures were prepared to validate the SLST assay. A fixed-composition community (FixedMix) contained 33.3% G1 and 16.7% each of A1, H2, K2 and L1. This mixture was used to evaluate quantitative accuracy of the assay on a non-even but constant composition. In addition, two staggered communities were generated to mimic highly imbalanced populations. StaggeredMix-L1 contained 92% L1 with ≤6% for the remaining SLST types, and StaggeredMix-G1 contained 96% G1 with ≤2% for the remaining types.

To assess assay robustness under conditions of low bacterial input and in the presence of background DNA, we generated a spike-in series based on an even five-SLST-type community composed of A1, G1, H2, K2 and L1 at 20% each. This community was supplemented with *Escherichia coli* genomic DNA (ATCC 8739D-5) to progressively reduce the fraction of *C. acnes* DNA. The resulting preparations contained final *C. acnes* fractions of 50% (SpikeMix-50), 10% (SpikeMix-10), 5% (SpikeMix-5) and 0.5% (SpikeMix-0.5), with *E. coli* DNA comprising the balance.

The theoretical composition of all mixes is presented in **Supplementary Table 1**. All mixtures were processed with the same SLST library preparation, sequencing and bioinformatic pipeline as the study samples.

Each defined mixture (FixedMix, StaggeredMix-L1 and StaggeredMix-G1) was prepared and sequenced in triplicate, and each spike-in mixture (SpikeMix-50, SpikeMix-10, SpikeMix-5 and SpikeMix-0.5) was processed in ten technical replicates. Replicates were processed independently through library preparation and sequencing, and replicate counts were combined within each mixture to generate pooled relative abundances for downstream analysis.

### DNA isolation

DNA extraction was performed upon sample reception, independently of the clinical center or groups. Until extraction, the samples were stored in 1 mL of DNA/RNA Shield (ZR1100, Zymo Research) at 4 °C. DNA was extracted using the ZymoBIOMICS DNA/RNA Mini kit (#R2002, Zymo Research), following the manufacturer’s instructions. Briefly, cells were disrupted using the BashingBead Lysis Tube (#S6003, Zymo Research) in a FastPrep-24 instrument (MP Biomedicals), for 3 × 40 sec at a speed setting of 6 (3 cycles were performed). The nucleic acids were eluted in 100 μL of elution buffer. Extracted DNA was then purified and concentrated using the Genomic DNA Clean & Concentrator-10 kit (#D4011, Zymo Research). Purified DNA was quantified using the QuantiFluor One dsDNA kit (#E4871, Promega) on the GloMax-Multi+ Detection System (Promega) (**Supplementary Table 5**).

### UMI-guided SLST library preparation

The SLST locus was sequenced using the LUMI-Seq synthetic long-read sequencing workflow, which enables accurate reconstruction of long amplicons from Illumina short reads through UMI–based molecule tracking. LUMI-Seq was originally developed for full-length 16S rRNA gene sequencing (IRT BIOASTER, 2021; Gravdal et al., 2023; Roume et al., 2023) and was adapted in this study to target the *C. acnes* SLST region (484 bp).

For clinical skin-strip samples, a pre-amplification of the SLST locus was performed prior to molecular barcoding, as the low bacterial DNA content of these samples (typically below 0.2 ng/µL total DNA; **Supplementary Table 5**) was insufficient to support adequate UMI redundancy in the barcoding step without prior enrichment. Pre-amplification was carried out using primers described by Petersen et al. (2017). Briefly, 5 µL of extracted DNA were combined with 11.75 µL of nuclease-free water and Kapa HiFi PCR reagents (Roche Diagnostics, #07958838001): 5 µL of 5× Kapa HiFi buffer, 0.75 µL of 100% DMSO, 0.75 µL of dNTP mix (10 mM), 0.5 µL of Kapa HiFi polymerase (1 U/µL), and 0.625 µL each of SLST-F and SLST-R primers (10 µM; primer sequences in **Supplementary Table 6**). PCR cycling conditions were 95 °C for 3 min, followed by 5 cycles of 95 °C for 30 s, 60 °C for 30 s, and 72 °C for 1 min, with a final hold at 4 °C. Amplified products were purified using AMPure XP beads (Beckman Coulter, #A63881) at a 0.6:1 bead-to-sample ratio.

Molecular barcoding was then performed to uniquely label individual SLST molecules using UMIs. For clinical samples, 5 µL of pre-amplified DNA were used, while mixes and controls were processed using 2 µL of purified DNA at 0.125 ng/µL. DNA was combined with nuclease-free water (8 or 11 µL, respectively) and Kapa2G PCR reagents (Roche Diagnostics, #07960760001): 5 µL of 5× Kapa2G buffer A, 5 µL of 5× enhancer buffer, 0.625 µL of dNTP mix (10 mM), 0.2 µL of Kapa2G polymerase (5 U), and 1.25 µL of SLST-SX-F-Pet primer (5 µM; **Supplementary Table 6**). A distinct primer was used for each sample, enabling simultaneous sample indexing and molecular barcoding. PCR cycling conditions were 95 °C for 3 min, followed by 2 cycles of 95 °C for 15 s, 50 °C for 20 s, and 72 °C for 2 min, with a final hold at 4 °C. Barcoded products were purified using AMPure XP beads at a 0.6:1 ratio.

To generate UMI redundancy and increase sequencing depth, barcoded molecules were subjected to an enrichment PCR. A total of 9 µL of purified barcoded DNA were combined with 7.75 µL of nuclease-free water and Kapa HiFi PCR reagents: 5 µL of 5× HiFi buffer, 0.75 µL of 100% DMSO, 0.75 µL of dNTP mix (10 mM), 0.5 µL of Kapa HiFi polymerase (1 U/µL), and 0.625 µL each of FLU primer and SLST-R-Pet primer (10 µM; **Supplementary Table 6**). PCR cycling conditions were 95 °C for 3 min, followed by 27 cycles of 95 °C for 30 s, 60 °C for 30 s, and 72 °C for 1 min, with a final hold at 4 °C. Enriched libraries were purified using AMPure XP beads at a 0.6:1 ratio and quantified using the QuantiFluor One dsDNA kit (Promega, #E4871) on a GloMax-Multi+ Detection System (Promega).

Following quantification, ten libraries were pooled equimolarly and subjected to Illumina library preparation using the Nextera XT DNA Library Preparation Kit (Illumina, #FC-131-1024) with minor modifications. Briefly, 5 µL of pooled DNA diluted to 0.2 ng/µL were mixed with 10 µL of TD buffer and 5 µL of ATM diluted 1:50, followed by incubation at 55 °C for 5 min. Tagmentation was stopped by adding 5 µL of NT buffer and incubating for 5 min at room temperature. PCR amplification was performed by adding 15 µL of NPM, 5 µL of N7XX index primer, and 5 µL of P5 primer (**Supplementary Table 6**), using the following cycling conditions: 72 °C for 3 min, 95 °C for 30 s, 14 cycles of 95 °C for 10 s, 55 °C for 30 s, and 72 °C for 30 s, followed by 72 °C for 5 min and a final hold at 4 °C. Libraries were purified twice using AMPure XP beads at a 0.6:1 ratio and quality-checked using the High Sensitivity NGS Fragment Analysis Kit (DNF-474, Agilent Technologies) on a Fragment Analyzer (Agilent Technologies) (**Supplementary Figure 4**).

### Sequencing

Sequencing was performed on a NextSeq500 system (Illumina) using the NextSeq 500/550 High Output Kit v2.5 (300 Cycles) in a 2×150 bp mode, in accordance with Illumina’s recommendations.

### Data processing

The data processing workflow is summarized in **Figure 1**. Raw BCL files were demultiplexed to per-sample FASTQ with bcl2fastq v2.20.0.422 (Illumina, https://support.illumina.com/sequencing/sequencing_software/bcl2fastq-conversion-software.html). Read quality was assessed with FastQC v0.11.5 (Andrews, 2010, https://www.bioinformatics.babraham.ac.uk/projects/fastqc/). Low-quality bases and Illumina adapters were trimmed with fastp v0.20.1 (Chen et al., 2018, https://github.com/OpenGene/fastp) using a 4-bp sliding window and a minimum mean Phred score of 20; reads <30 bp after trimming were discarded. Unpaired mates were removed with BBTools “repair.sh” v38.79 (Bushnell, 2014, https://github.com/bbushnell/BBTools).

For single-locus sequence typing (SLST), reads were partitioned by sample and by unique molecular identifier (UMI) into barcode families. Families supported by ≥50 reads were assembled *de novo* with SPAdes v3.14.1 (Prjibelski et al., 2020, https://github.com/ablab/spades) based on background levels observed in negative controls. The largest contig from each family was screened for a full-length SLST locus using nhmmscan (HMMER v3.3.2; Eddy, 2011, http://hmmer.org/) against profile HMMs built from 161 SLST sequences downloaded from the *C. acnes* SLST database on 22 May 2020 (Scholz et al., 2014). Full-length SLST sequences were assigned to SLST types by blastn v2.12.0+ (Camacho et al., 2009, https://ftp.ncbi.nlm.nih.gov/blast/executables/blast+/LATEST/) against the *C. acnes* SLST database (226 sequences, updated 28 February 2025), requiring 100% identity and 100% coverage.

All downstream summaries and visualizations were performed in R v4.3.3 (R Core Team, 2024) using ggplot2 (https://github.com/tidyverse/ggplot2) for plotting and the tidyverse packages for data wrangling (https://github.com/tidyverse), plus ggrepel (https://github.com/slowkow/ggrepel), RColorBrewer (https://cran.r-project.org/package=RColorBrewer), and patchwork (https://github.com/thomasp85/patchwork).

### Application of the SLST assay to skin samples and controls

Clinical skin-strip DNAs were processed using the validated SLST workflow. Each sequencing run included an in-house positive control (“Mock”) composed of six SLST types (A1, F4, G1, H2, K2 and L1) combined at equal nominal proportions and spiked with *E. coli* genomic DNA (ATCC 8739D-5) at a 2:98 mass ratio quantified with QuantiFluor One. This control was sequenced alongside all skin samples to monitor assay performance and ensure run-to-run consistency.

Each run also included negative controls consisting of nuclease-free water processed through the entire workflow in parallel with the samples. These controls were used to monitor potential contamination and to define a threshold for background noise.

### Phylotyping by multiplex PCR

To compare *C. acnes* phylogroup composition obtained by SLST sequencing with a culture-based approach, selected skin-strip samples were analyzed by isolation followed by multiplex PCR phylotyping, using the assay described by Barnard et al. (2015).

Isolates originated from gel strip samples collected on the back. Each strip was vortexed in phosphate-buffered saline (PBS) to release bacteria, serially diluted, and plated on Columbia agar supplemented with 5% horse blood. Plates were incubated under anaerobic conditions at 37 °C for 5–7 days.

From each sample, up to 45 single colonies displaying a *C. acnes*-like morphology were randomly selected. Colonies were initially screened for species identification using *C. acnes*-specific 16S rRNA primers (PArA-1 and PArA-2; **Supplementary Table 7**). PCR-positive colonies were cultured in GAMm medium, and genomic DNA was extracted using the PureLink™ Microbiome DNA Purification Kit (Thermo Fisher Scientific, A29790).

Phylogroup assignment was performed using a multiplex PCR assay targeting phylogroup-discriminatory genes. Primer pairs targeted the 16S rRNA gene (species confirmation), *atpA* (ATPase; phylogroups IA1, IA2, IC), *sodA* (IA2, IB), *fic* (fic toxin; IC), *atpD* (II), and *recA* (III). Primer sequences, gene targets, and phylogroup specificities are provided in **Supplementary Table 7**. Primers were pooled at a final concentration of 0.2 µM each and amplified using Platinum™ Multiplex Taq DNA Polymerase (Thermo Fisher Scientific, 4464268).

For each sample, phylogroup relative abundances were calculated as the proportion of colonies assigned to each phylogroup by multiplex PCR.

## Data Availability

The datasets generated during the current study are available on DDBJ/EMBL/GenBank as a Targeted Locus Study project (BioProject ID PRJNA1422617) under accession KJKS00000000. The version described in this paper is KJKS01000000.

## Supplementary material

Supplementary Figure 1 Schematic overview of the SLST-Seq workflow. The diagram illustrates the main steps of the UMI-based synthetic long-read sequencing approach applied to the *Cutibacterium acnes* SLST locus, including molecular barcoding of individual SLST molecules, amplification, short-read sequencing, grouping of reads by unique molecular identifiers (UMIs), and reconstruction of full-length SLST sequences.

Supplementary Figure 2 Run-to-run performance of the SLST assay on an internal Mock control. **(A)** Relative abundances of the six SLST types (A1, F4, G1, H2, K2 and L1) recovered from the Mock control included in each sequencing run. The Mock sample consisted of equal nominal proportions of the six SLST types spiked with *E. coli* DNA (2:98 mass ratio). **(B)** Percentage of reads per run assigned to sequences other than the six target SLST types (“Others”) or not assigned to any SLST type (“Unassigned”). The Mock control yielded stable relative abundances and low background across eleven independent sequencing runs. Average values and variability across runs are provided in **Table 2** and **Supplementary Table 2**.

Supplementary Figure 3 Correlation between multiplex PCR and SLST phylotype abundances across shared skin samples. Each panel corresponds to one *Cutibacterium acnes* phylotype, showing the relative abundance (%) obtained by multiplex PCR (x-axis) and by SLST (y-axis). The dashed line indicates the 1:1 identity line, and the solid line shows the linear regression fit. The Pearson correlation coefficient (*r*) and the mean absolute error (MAE, in percentage points) are indicated in each panel.

Supplementary Figure 4 Quality control profiles of SLST-Seq libraries based on fragment size distribution. **(A)** Representative SLST library after enrichment PCR. **(B)** Pooled SLST library prior to Illumina sequencing. Vertical blue lines indicate the target fragment size range used for library validation.

Supplementary Table 1 Theoretical composition (%) of SLST types in the defined mock communities used to validate the assay.

Supplementary Table 2 Summary statistics of observed relative abundances for the six SLST types in the Mock control across 12 sequencing runs. For each type, the expected proportion (16.7%) is compared with the mean observed proportion, standard deviation (SD), and range (minimum–maximum).

Supplementary Table 3 Full-length SLST sequences recovered from negative controls (nuclease-free water) included in each of the twelve sequencing runs.

Supplementary Table 4 Bray-Curtis similarity between observed and expected SLST type compositions across the SpikeMix dilution series. Ten technical replicates were processed per condition. Bray-Curtis similarity was computed between observed relative abundances of the five target SLST types (A1, H2, G1, K2, L1) and the expected equal composition (20% each).

Supplementary Table 5 Quality and quantity of extracted DNA.

Supplementary Table 6 Primer sequences used for SLST amplification, UMI-based molecular barcoding, and Illumina library preparation.

Supplementary Table 7 Primer sequences and targets used for *Cutibacterium acnes* species identification and phylogroup assignment by multiplex PCR. Adapted from the phylotyping scheme of Barnard et al. (2015).

## References

[B1] Ahle C. M. Stødkilde K. Poehlein A. Bömeke M. Streit W. R. Wenck H. . (2022). Interference and co-existence of staphylococci and Cutibacterium acnes within the healthy human skin microbiome. Commun. Biol. 5, 923. doi: 10.1038/s42003-022-03897-6. PMID: 36071129 PMC9452508

[B2] Andrews S. (2010). FastQC: A Quality Control Tool for High Throughput Sequence Data. Available online at: https://www.bioinformatics.babraham.ac.uk/projects/fastqc/ (Accessed April 30, 2026).

[B3] Barnard E. Nagy I. Hunyadkürti J. Patrick S. McDowell A. (2015). Multiplex touchdown PCR for rapid typing of the opportunistic pathogen propionibacterium acnes. J. Clin. Microbiol. 53, 1149–1155. doi: 10.1128/jcm.02460-14. PMID: 25631794 PMC4365214

[B4] Brüggemann H. Salar-Vidal L. Gollnick H. P. M. Lood R. (2021). A Janus-faced bacterium: Host-beneficial and -detrimental roles of Cutibacterium acnes. Front. Microbiol. 12. doi: 10.3389/fmicb.2021.673845. PMID: 34135880 PMC8200545

[B5] Bushnell B. (2014). BBMap: A Fast, Accurate, Splice-Aware Aligner. Available online at: https://bbmap.org (Accessed April 30, 2026).

[B6] Camacho C. Coulouris G. Avagyan V. Ma N. Papadopoulos J. Bealer K. . (2009). BLAST+: architecture and applications. BMC Bioinf. 10, 421. doi: 10.1186/1471-2105-10-421. PMID: 20003500 PMC2803857

[B8] Chen Y. Knight R. Gallo R. L. (2023). Evolving approaches to profiling the microbiome in skin disease. Front. Immunol. 14. doi: 10.3389/fimmu.2023.1151527. PMID: 37081873 PMC10110978

[B7] Chen S. Zhou Y. Chen Y. Gu J. (2018). fastp: an ultra-fast all-in-one FASTQ preprocessor. Bioinformatics 34, i884–i890. doi: 10.1093/bioinformatics/bty560. PMID: 30423086 PMC6129281

[B9] Cobian N. Garlet A. Hidalgo-Cantabrana C. Barrangou R. (2021). Comparative genomic analyses and CRISPR-Cas characterization of Cutibacterium acnes provide insights into genetic diversity and typing applications. Front. Microbiol. 12. doi: 10.3389/fmicb.2021.758749. PMID: 34803983 PMC8595920

[B10] Dagnelie M.-A. Corvec S. Saint-Jean M. Bourdès V. Nguyen J.-M. Khammari A. . (2018a). Decrease in diversity of Propionibacterium acnes phylotypes in patients with severe acne on the back. Acta Dermato-Venereologica. 98, 262–267. doi: 10.2340/00015555-2847. PMID: 29136261

[B11] Dagnelie M.-A. Khammari A. Dréno B. Corvec S. (2018b). Cutibacterium acnes molecular typing: time to standardize the method. Clin. Microbiol. Infection. 24, 1149–1155. doi: 10.1016/j.cmi.2018.03.010. PMID: 29544912

[B12] Dekio I. Asahina A. Shah H. N. (2021). Unravelling the eco-specificity and pathophysiological properties of Cutibacterium species in the light of recent taxonomic changes. Anaerobe 71, 102411. doi: 10.1016/j.anaerobe.2021.102411. PMID: 34265438

[B13] Eddy S. R. (2011). Accelerated profile HMM searches. PloS Comput. Biol. 7, e1002195. doi: 10.1371/journal.pcbi.1002195. PMID: 22039361 PMC3197634

[B14] Feidenhansl C. Lund M. Poehlein A. Lood R. Lomholt H. B. Brüggemann H. (2024). Cutibacterium and Staphylococcus dysbiosis of the skin microbiome in acne and its decline after isotretinoin treatment. JEADV. Clin. Pract. 3, 1454–1466. doi: 10.1002/jvc2.487. PMID: 41531421

[B15] Gravdal K. Kirste K. H. Grzelak K. Kirubakaran G. T. Leissner P. Saliou A. . (2023). Exploring the gut microbiota in patients with pre-diabetes and treatment naïve diabetes type 2 - a pilot study. BMC Endocr. Disord. 23, 179. doi: 10.1186/s12902-023-01432-0. PMID: 37605183 PMC10440924

[B16] IRT BIOASTER (2021). LUMI-Seq®: Technologies Designed by BIOASTER. Available online at: https://www.youtube.com/watch?v=-fAhmEVzFKY (Accessed April 30, 2026).

[B17] Lomholt H. B. Kilian M. (2010). Population genetic analysis of Propionibacterium acnes identifies a subpopulation and epidemic clones associated with acne. PloS One 5, e12277. doi: 10.1371/journal.pone.0012277. PMID: 20808860 PMC2924382

[B18] Markey L. Qu E. B. Mendall C. Finzel A. Materna A. Lieberman T. D. (2025). Microbiome diversity of low biomass skin sites is captured by metagenomics but not 16S amplicon sequencing 2025.06.24.661265. doi: 10.1101/2025.06.24.661265

[B19] McDowell A. Gao A. Barnard E. Fink C. Murray P. I. Dowson C. G. . (2011). A novel multilocus sequence typing scheme for the opportunistic pathogen Propionibacterium acnes and characterization of type I cell surface-associated antigens. Microbiology 157, 1990–2003. doi: 10.1099/mic.0.049676-0. PMID: 21511767

[B20] Petersen R. L. W. Scholz C. F. P. Jensen A. Brüggemann H. Lomholt H. B. (2017). Propionibacterium acnes phylogenetic type III is associated with progressive macular hypomelanosis. Eur. J. Microbiol. Immunol. 7, 37–45. doi: 10.1556/1886.2016.00040. PMID: 28386469 PMC5372479

[B21] Prjibelski A. Antipov D. Meleshko D. Lapidus A. Korobeynikov A. (2020). Using SPAdes De novo assembler. Curr. Protoc. Bioinf. 70, e102. doi: 10.1002/cpbi.102. PMID: 32559359

[B22] R Core Team (2024). R: A Language and Environment for Statistical Computing (Vienna, Austria: R Foundation for Statistical Computing). Available online at: https://www.R-project.org (Accessed April 30, 2026).

[B23] Roume H. Mondot S. Saliou A. Le Fresne-Languille S. Doré J. (2023). Multicenter evaluation of gut microbiome profiling by next-generation sequencing reveals major biases in partial-length metabarcoding approach. Sci. Rep. 13, 22593. doi: 10.1038/s41598-023-46062-7. PMID: 38114587 PMC10730622

[B24] Scholz C. F. P. Jensen A. Lomholt H. B. Brüggemann H. Kilian M. (2014). A novel high-resolution single locus sequence typing scheme for mixed populations of Propionibacterium acnes *in vivo*. PloS One 9, e104199. doi: 10.1371/journal.pone.0104199. PMID: 25111794 PMC4128656

[B25] Spittaels K.-J. Ongena R. Zouboulis C. C. Crabbé A. Coenye T. (2020). Cutibacterium acnes phylotype I and II strains interact differently with human skin cells. Front. Cell. Infect. Microbiol. 10. doi: 10.3389/fcimb.2020.575164. PMID: 33330124 PMC7717938

[B26] Yu T. Xu X. Liu Y. Wang X. Wu S. Qiu Z. . (2024). Multi-omics signatures reveal genomic and functional heterogeneity of Cutibacterium acnes in normal and diseased skin. Cell. Host Microbe 32, 1129–1146.e8. doi: 10.1016/j.chom.2024.06.002. PMID: 38936370

[B27] Zhang H. Wang X. Chen A. Li S. Tao R. Chen K. . (2024). Comparison of the full-length sequence and sub-regions of 16S rRNA gene for skin microbiome profiling. mSystems 9, e00399-24. doi: 10.1128/msystems.00399-24. PMID: 38934545 PMC11264597

