# Supplementary figures and images for: UMI-guided single locus sequence typing method for phylotyping Cutibacterium acnes from skin samples
Source: Front Cell Infect Microbiol. 2026 May 20;16:1807759. doi: 10.3389/fcimb.2026.1807759 (PMC13231282; doi:10.3389/fcimb.2026.1807759)

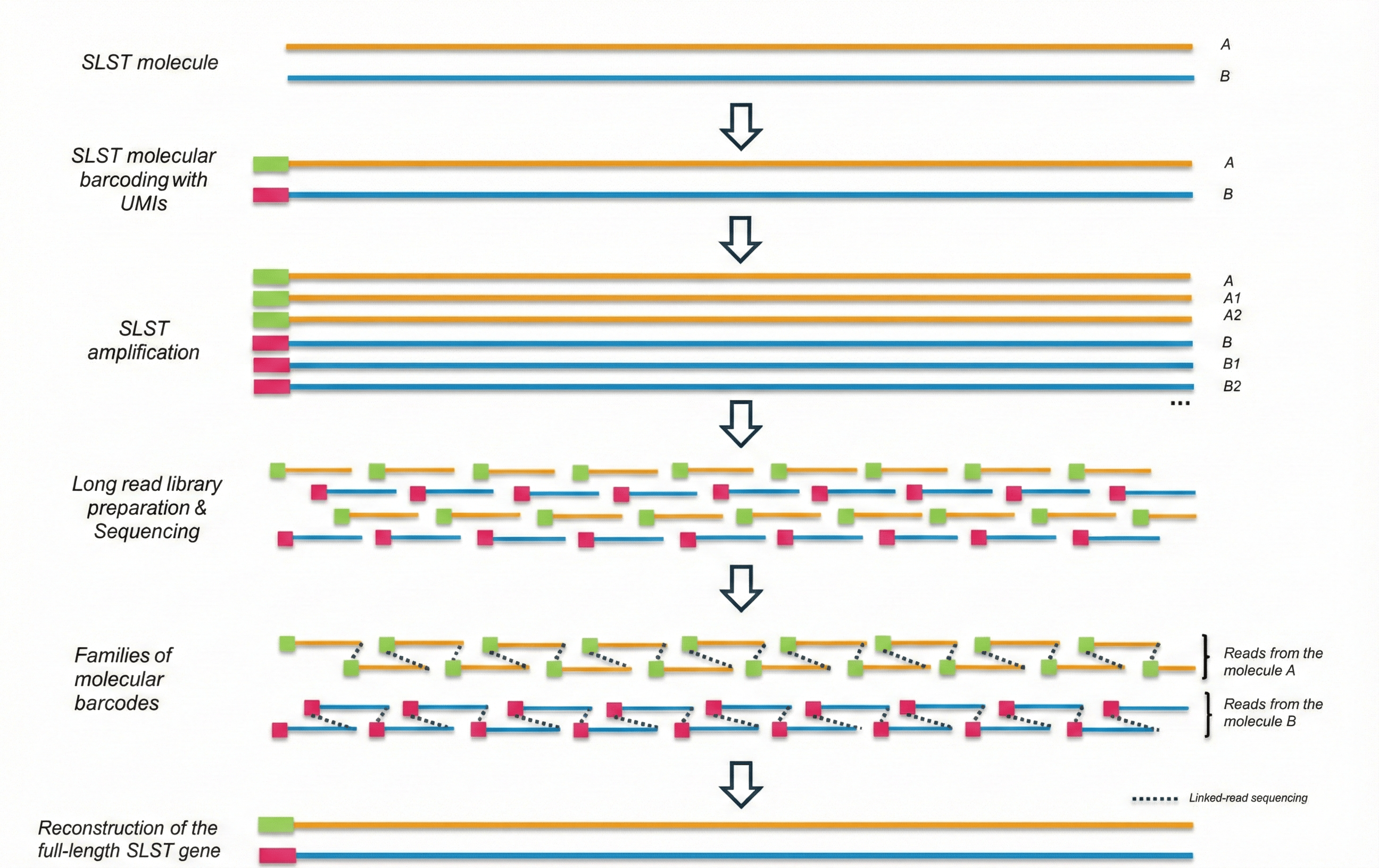

Supplement: Supplementary file 8 [file Image1.tiff]

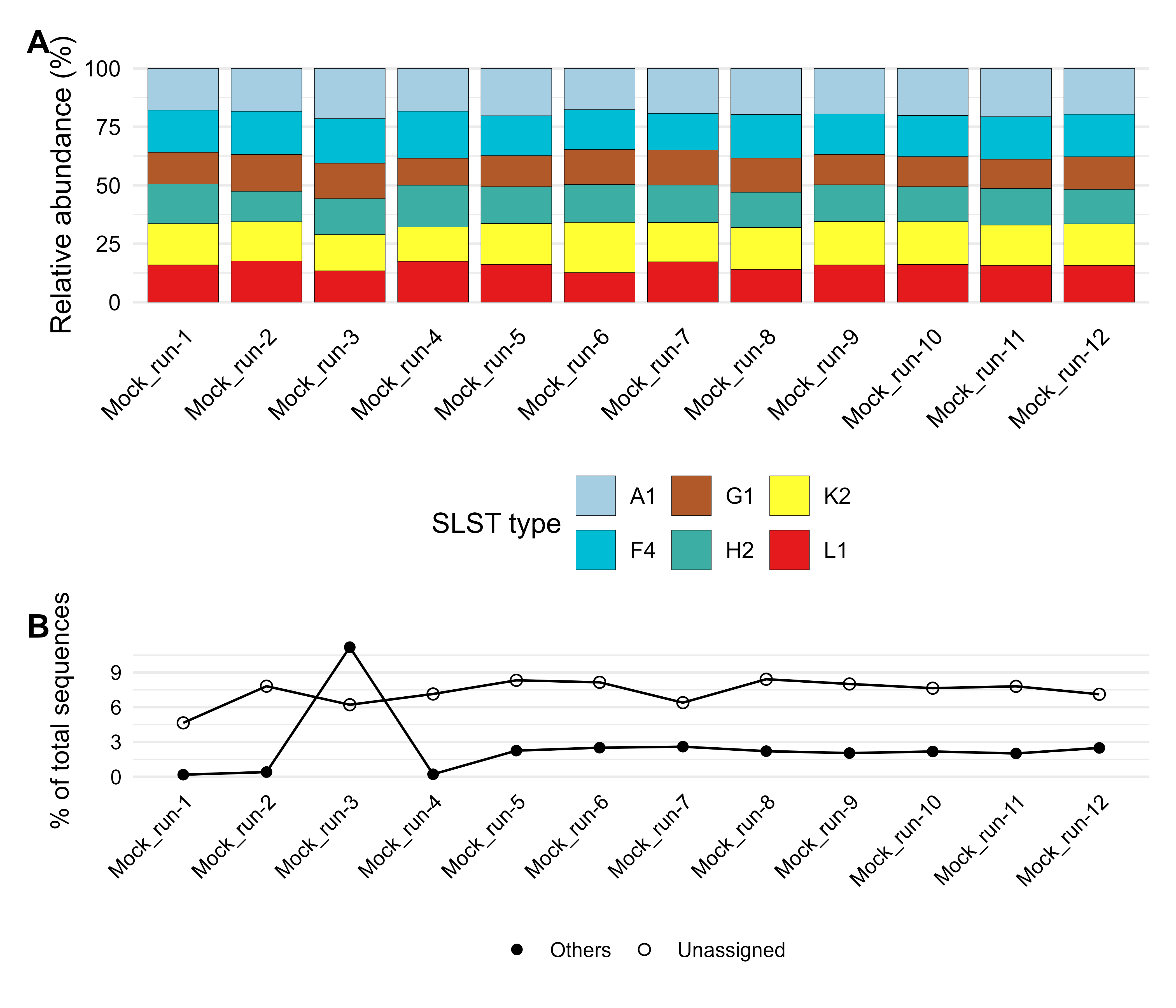

Supplement: Supplementary file 9 [file Image2.tiff]

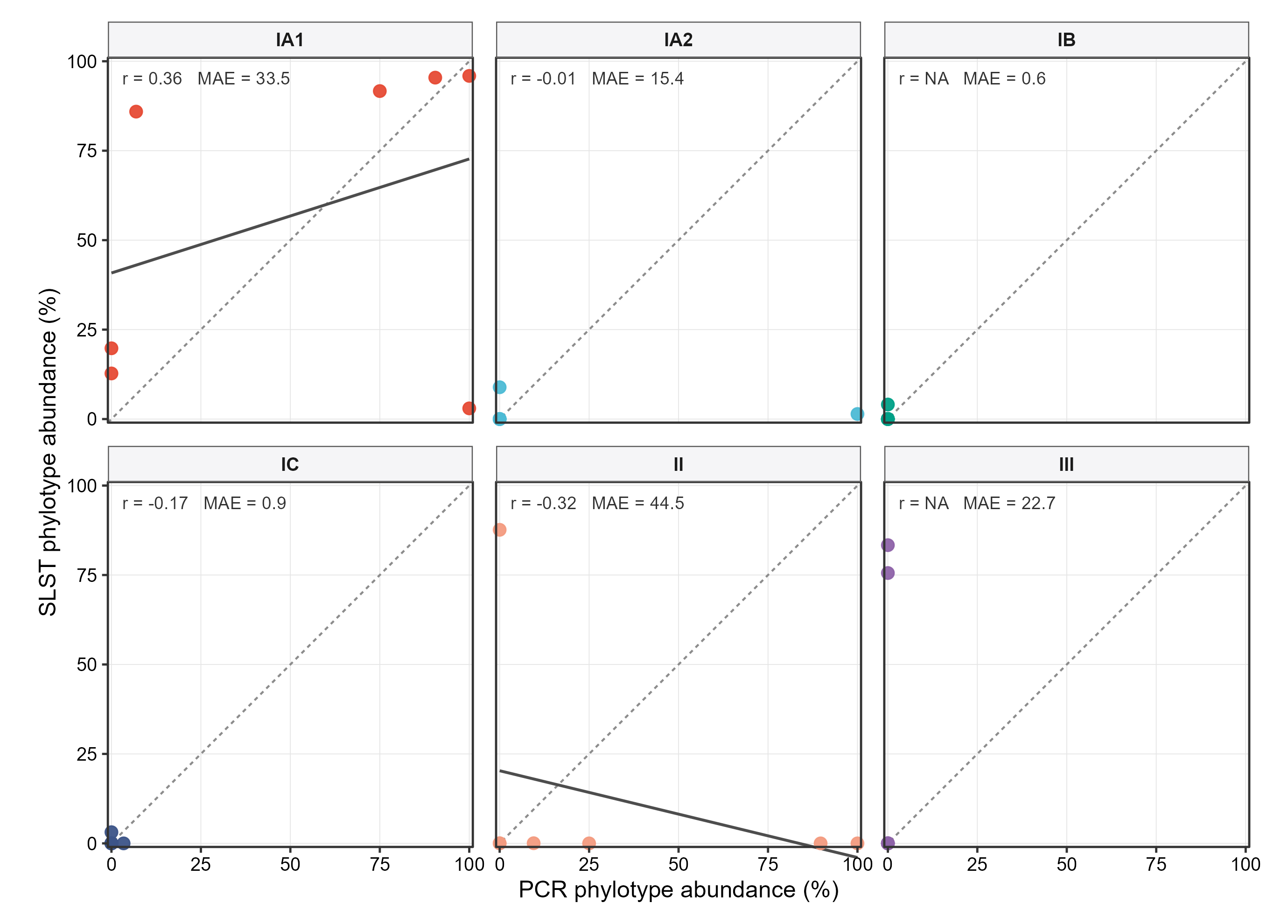

Supplement: Supplementary file 10 [file Image3.tiff]

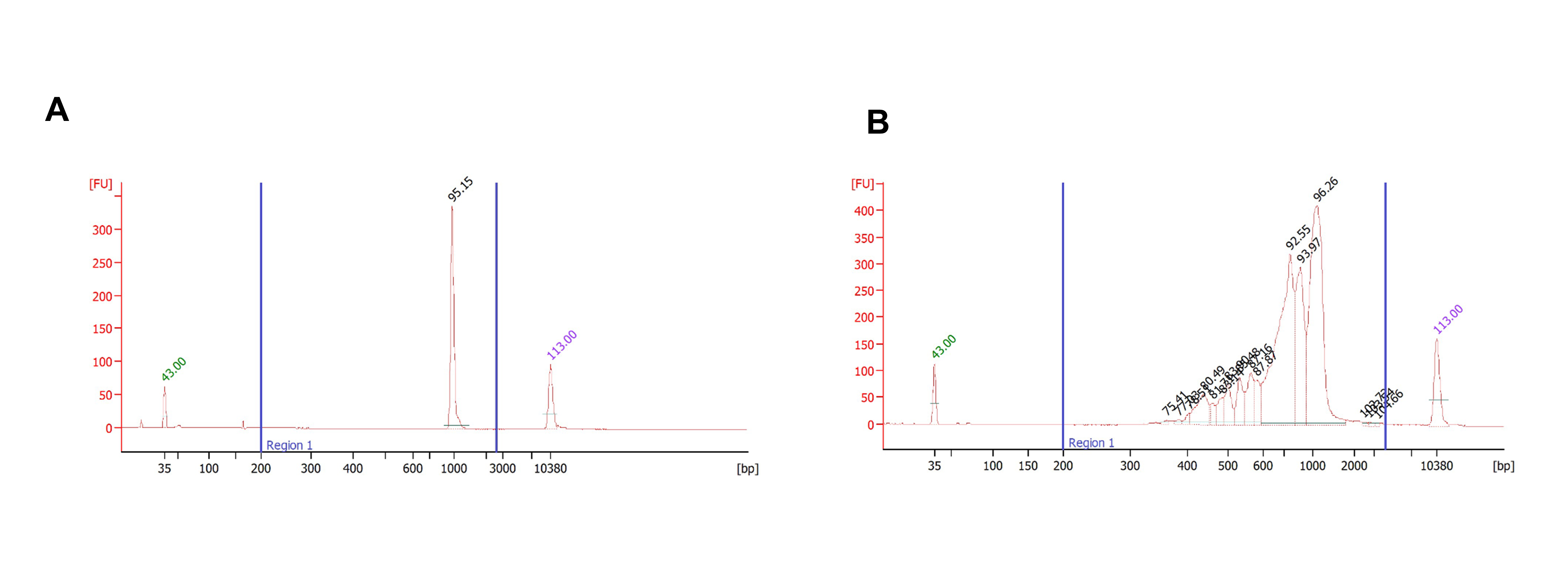

Supplement: Supplementary file 11 [file Image4.tiff]
